# Brain pharmacokinetics of mono- and bispecific amyloid-β antibodies in wild-type and Alzheimer’s disease mice measured by high cut-off microdialysis

**DOI:** 10.1186/s12987-022-00398-w

**Published:** 2022-12-12

**Authors:** Ulrika Julku, Mengfei Xiong, Elin Wik, Sahar Roshanbin, Dag Sehlin, Stina Syvänen

**Affiliations:** Rudbeck Laboratory, Department of Public Health and Caring Sciences, Uppsala University, Dag Hammarskjölds Väg 20, 751 85 Uppsala, Sweden

**Keywords:** Bispecific antibody, Amyloid-β, Transferrin receptor, Microdialysis, Blood–brain barrier

## Abstract

**Background:**

Treatment with amyloid-β (Aβ) targeting antibodies is a promising approach to remove Aβ brain pathology in Alzheimer's disease (AD) and possibly even slow down or stop progression of the disease. One of the main challenges of brain immunotherapy is the restricted delivery of antibodies to the brain. However, bispecific antibodies that utilize the transferrin receptor (TfR) as a shuttle for transport across the blood–brain barrier (BBB) can access the brain better than traditional monospecific antibodies. Previous studies have shown that bispecific Aβ targeting antibodies have higher brain distribution, and can remove Aβ pathology more efficiently than monospecific antibodies. Yet, there is only limited information available on brain pharmacokinetics, especially regarding differences between mono- and bispecific antibodies.

**Methods:**

The aim of the study was to compare brain pharmacokinetics of Aβ-targeting monospecific mAb3D6 and its bispecific version mAb3D6-scFv8D3 that also targets TfR. High cut-off microdialysis was used to measure intravenously injected radiolabelled mAb3D6 and mAb3D6-scFv8D3 antibodies in the interstitial fluid (ISF) of hippocampus in wild-type mice and the *App*^*NL−G−F*^ mouse model of AD. Distribution of the antibodies in the brain and the peripheral tissue was examined by ex vivo autoradiography and biodistribution studies.

**Results:**

Brain concentrations of the bispecific antibody were elevated compared to the monospecific antibody in the hippocampal ISF measured by microdialysis and in the brain tissue at 4–6 h after an intravenous injection. The concentration of the bispecific antibody was approximately twofold higher in the ISF dialysate compared to the concentration of monospecific antibody and eightfold higher in brain tissue 6 h post-injection. The ISF dialysate concentrations for both antibodies were similar in both wild-type and *App*^*NL−G−F*^ mice 24 h post-injection, although the total brain tissue concentration of the bispecific antibody was higher than that of the monospecific antibody at this time point. Some accumulation of radioactivity around the probe area was observed especially for the monospecific antibody indicating that the probe compromised the BBB to some extent at the probe insertion site.

**Conclusion:**

The BBB-penetrating bispecific antibody displayed higher ISF concentrations than the monospecific antibody. The concentration difference between the two antibodies was even larger in the whole brain than in the ISF. Further, the bispecific antibody, but not the monospecific antibody, displayed higher total brain concentrations than ISF concentrations, indicating association to brain tissue.

**Supplementary Information:**

The online version contains supplementary material available at 10.1186/s12987-022-00398-w.

## Background

Antibodies and other biologics are increasingly used as therapeutics not only for peripheral diseases but also as treatments for central nervous system (CNS) disorders. For example, the first disease-modifying treatment for Alzheimer's disease (AD), the most common dementia disorder, is an antibody directed towards amyloid-beta (Aβ). This antibody, *aducanumab* [1, 2], was conditionally approved in 2021 by the US Food and Drug Administration. Additionally, three anti-Aβ antibodies (*lecanemab* [3, 4], *gantenerumab* [5, 6] and *donanemab* [7, 8]) are presently studied in phase III clinical trials. Antibodies are large molecules and therefore display very limited passage across the blood–brain barrier (BBB). It is estimated that less than 1 in 1000 antibody molecules reach the brain, as several studies report brain antibody concentrations of less than 0.1% of the injected dose [9–12]. As a strategy to increase the fraction of administered antibody that can pass the BBB, antibodies fused to an additional binding moiety directed towards the transferrin receptor (TfR) have been designed. The TfR is expressed by the endothelial cells of the BBB, and proteins binding to TfR may be shuttled into the brain by receptor-mediated transcytosis. Thus, bispecific antibodies that bind to both TfR and Aβ display 10- to 100-fold higher brain concentrations than monospecific (unmodified) antibodies [13–17]. One such bispecific antibody, based on *gantenerumab*, has already entered phase I clinical trials [18]. Despite the use of monospecific antibodies in AD patients, and the emergence of bispecific antibody versions, very little is known about their brain pharmacokinetics in terms of brain entry, intrabrain distribution and elimination. Most studies of antibody brain pharmacokinetics report total brain concentrations, or CSF concentrations, at discrete time points [1, 3]. It appears that monospecific antibodies enter the brain more slowly, while bispecific antibodies with a high affinity towards the TfR display a concentration maximum in the brain already within an hour, or perhaps within minutes, after administration at least if dosed at sub-pharmacological doses [11, 19]. However, antibody concentrations should be measured continuously over an extended time to fully describe the time-aspects of brain entry and distribution. There are only a few methods that allow for this. Multiphoton imaging has been used to follow the distribution of fluorophore-labelled antibodies and antibody-fragments from the brain vasculature into the brain parenchyma [20]. Although, it is very informative for comparison of proteins in terms of temporal BBB passage and distance of diffusion within the brain parenchyma, it provides mainly qualitative rather than quantitative information. Medical imaging methods, e.g. positron emission tomography (PET), can be used to monitor brain concentrations of radiolabelled molecules, including radiolabelled antibodies [21–23]. However, for antibodies that show very limited brain delivery, the signal originating from labelled antibodies residing in the blood volume of the brain, which is approximately 5% of the brain volume, may mask the signal from antibodies that are present in the brain parenchyma. Further, PET cannot distinguish between unbound and bound molecules, or between extra- and intracellular concentrations. One method that enables investigations of unbound drug concentrations in the brain interstitial fluid (ISF) over an extended period of time is microdialysis. Microdialysis is based on the surgical insertion of a semipermeable probe into the tissue of interest [24, 25]. The technique has mainly been used for studies of small molecular drugs, but recent development of high cut-off probe membranes has allowed the measurement of proteins in tissues, including brain tissue [26]. For example, the technique has been used to measure endogenous proteins such as Aβ and tau [27, 28]. Microdialysis studies have shown that ISF concentration of Aβ and Tau are elevated in the brain of AD mice [28, 29] and Aβ has shown to have diurnal variation in the ISF [27]. A few studies have also used microdialysis to measure pharmacokinetics of intravenously administered proteins in the mouse or rat brain [30–33].

The aim of the present study was to compare brain pharmacokinetics of Aβ-targeting monospecific mAb3D6 and bispecific mAb3D6-scFv8D3 antibodies in wild-type (Wt) and AD mice (*App*^*NL−G−F*^). The 3D6 antibody was selected due to its ability to detect all forms of Aβ, irrespectively of antibody format [34]. The antibody concentration in the brain ISF was studied by high cut-off microdialysis, and the antibody distribution in the brain tissue and in peripheral tissues were studied by ex vivo autoradiography and biodistribution.

## Methods

### Antibodies

The two antibodies, monospecific mAb3D6 and bispecific mAb3D6-scFv8D3 were cloned, expressed and purified by affinity chromatography according to a previously published protocol [35]. After purification, antibodies were aliquoted and stored in − 70 °C until use.

### Radiochemistry

The antibodies, mAb3D6 and mAb3D6-scFv8D3, were labeled with iodine-125 (^125^I) by direct iodination with chloramine T [36]. Briefly, antibody (mAb3D6 or mAb3D6-scFv8D3), ^125^I stock solution (Perkin Elmer, USA) and chloramine T (5 µg) were mixed in PBS to a final volume of 110 μL, and then incubated 90 s in room temperature. The labeling reaction was quenched with 10 μg sodium metabisulfite. Radioiodinated antibody was purified with Zeba Spin Desalting Columns (7 K MWCO, 0.5 mL, Thermo Fisher Scientific, Waltham, MA, USA). Binding of [^125^I]I-mAb3D6 and [^125^I]I-mAb3D6-scFv8D3 to Aβ and TfR was tested with ELISA directly after radiolabeling according to a previously described method [16].

### Animals

Wild-type (Wt) C57BL/6JBomTac (n = 17) and *App*^*NL−G−F*^ mice (n = 8) at the age of 8 months were used in the experiments. *App*^*NL−G−F*^ is a single *APP* knock-in mouse model harboring the Swedish (KM670/671NL), Arctic (E693G) and Beyreuter/Iberian (I716F) *APP* mutations [37]. *App*^*NL−G−F*^ mice are characterized by rapidly evolving Aβ42 pathology in the brain. Plaque pathology is first visible at the age of 3–4 months and abundant at the age of 8 months, i.e. the age at which mice were investigated in the present study.

The mice were housed in animal facility at Uppsala University in individually ventilated cages with 12/12 h dark–light cycle and ad libitum access to food pellets and tap water. All animal experiments were approved by the Uppsala County Animal Ethics board (5.8.18-20401-2020) following the legislation and regulations of the Swedish Animal Welfare Agency and European Communities Council Directive of 22 September 2010 (20103/EU).

### Surgery

A guide cannula (AT12.8.iC, AgnTho's, Lidingö, Sweden) was inserted into the left hippocampus (coordinates A/P + 2.2, M/L + 1.2 from bregma, and D/V − 1.5 from dura) by stereotaxic surgery under isoflurane anesthesia (induction 4% and maintenance 2%; Isofluran Baxter, Baxter S.A., Lessines, Belgium). The cannula was secured on the skull with two anchor screws (1 × 2 mm, AgnTho's) and dental cement (Dentalon plus, Heraeus Kulzer GmbH, Hanau, Germany). Buprenorphine (Bupaq vet, Richter Pharma AG, Wels, Austria) and meloxicam (Metacam, Boehringer Ingelheim Vetmedica GMBH, Rohrdorf, Germany) were administered subcutaneously for post-operative pain and lidocaine (Xylocain, Aspen Pharma Trading Ltd, Dublin, Ireland) was used as a local anesthetic. Mice were allowed to recover 8–10 days before the microdialysis.

### Microdialysis

Prior to the microdialysis, fluorinated ethylene propylene tubing (FEP PTFE tubing, ID 0.12 mm, AgnTho's), FEP Tubing Connector Peristaltic Kit (CMA Microdialysis AB, Kista, Sweden), and the probe (AT12.8.1, 1 mm PE membrane, 3 MDa cut-off, AgnTho's) were coated with 5% PEI (Polyethyleneimine MW ~ 2000, Sigma Aldrich, Saint Louis, MO, USA), 0.5 μL/min for 16 h to prevent binding of the radiolabeled antibody to the tubing and probe and to improve probe recovery during the microdialysis as described in [38, 39]. After the coating, tubing and probe were washed with water, 10 μL/min for 10 min, then 1 μL/min for 8 h. The connections of the FEP tubing were secured with Tygon tubing R3607 ID 0.38 mm (Ismatec, Cole-Parmer GmbH, Wertheim, Germany) and the connection between FEP tubing and probe inlet or outlet with Tygon tubing R3607 ID 0.25 mm (Ismatec).

**Fig. 1:**
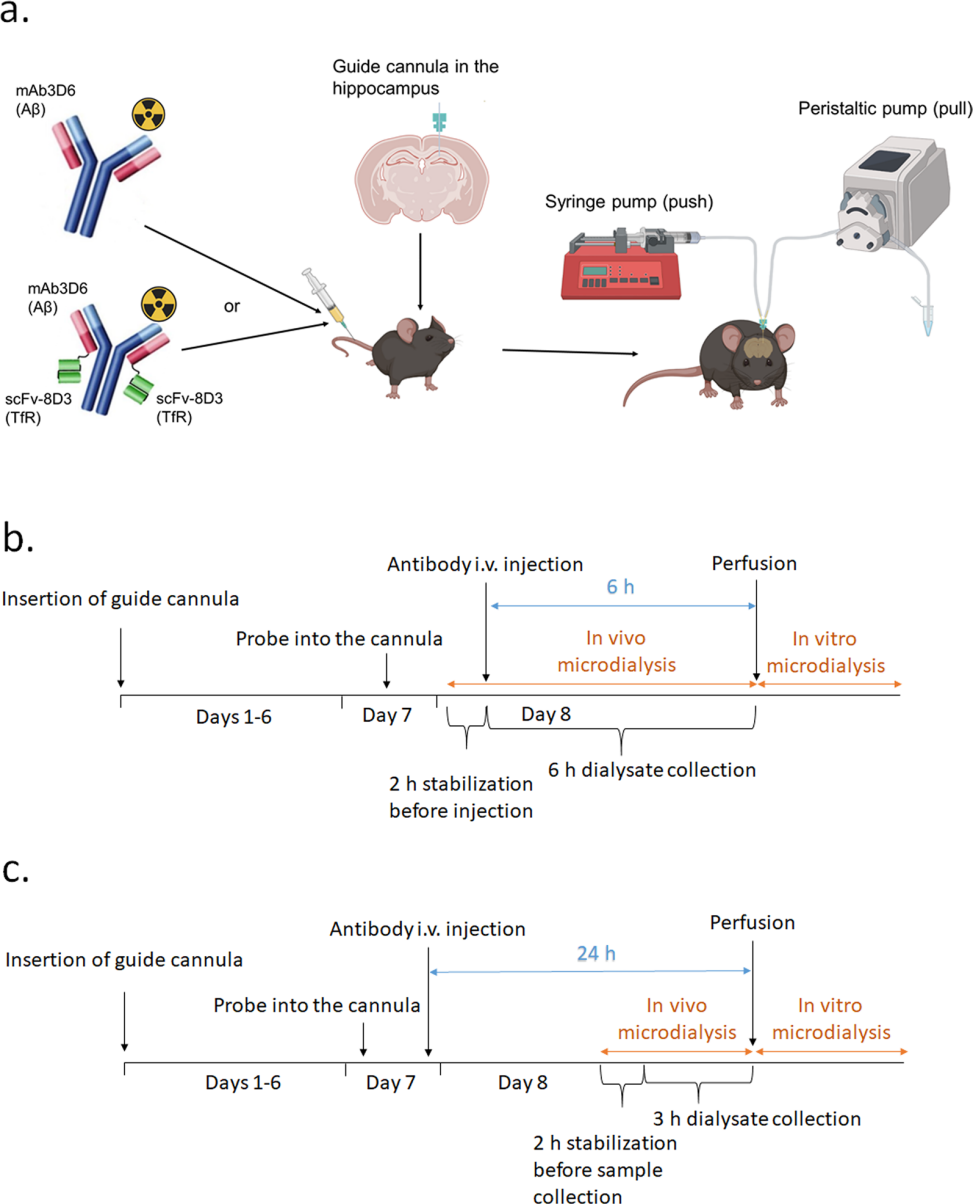
Intravenously injected monospecific [^125^I]I-mAb3D6 or bispecific [^125^I]I-mAb3D6-scFv8D3 were measured in the ISF of hippocampus using push-and-pull microdialysis. **a** Timeline for the microdialysis experiments 6 h, **b** or 24 h, **c** post-injection. Part of figure a. was made in BioRender

The probe was inserted into the guide cannula the day before the microdialysis experiment. On the morning of the microdialysis, mice were placed into the Rotating Animal Cage System (RACS, AgnTho's), and the probe was connected to the microdialysis tubing and push-and-pull microdialysis setup containing CMA 402 Microdialysis syringe pump (CMA Microdialysis AB), Reglo ICC Digital Peristaltic pump (CMA Microdialysis AB) and CMA 470 Refrigerated Microfraction Collector (CMA Microdialysis AB) (Fig. 1a). The probe was perfused at 0.5 μL/min with Ringer solution containing 0.15% BSA starting 2 h before antibody injection or 2 h before start of collection for the late time-point (21–24 h) to stabilize the probe. Mice were briefly anesthetized with isoflurane and ^125^I-labeled mAb3D6 (6.5 nmol/kg, 5.07 ± 0.45 MBq) or mAb3D6-scFv8D3 (6.5 nmol/kg, 5.03 ± 0.38 MBq) was intravenously administered in the tail vein. Dialysate was collected for 6 h post-injection (12 × 30 min) in Wt mice (Fig. 1b) or 21–24 h post-injection (6 × 30 min) in Wt and App^NL−G−F^ mice (Fig. 1c) into 300 μL polypropylene vials (AgnTho's). The reported 24-h microdialysis results were calculated as the average of the samples collected 21–24 h post-injection. The volume of the dialysate was measured by weighing the samples immediately after collection, and peristaltic pump flow was adjusted, based on the fluid recovery, if the fluid recovery was lower or higher than 97–103% as described in [30]. As a control, the brain distribution of the radiolabeled antibodies was compared with the distribution of radiolabel itself. This was done by injecting mice with ^125^I (5.21 ± 0.16 MBq) in PBS according to the same procedure as described for the antibodies. Following the microdialysis, a transcardial perfusion with saline was performed under terminal isoflurane anesthesia (3%) to remove blood from the tissue. A terminal blood sample from heart was collected before the perfusion, and the brain was isolated after the perfusion.

In vitro microdialysis was performed following the in vivo microdialysis to measure in vitro probe recovery. The probe was removed from the brain and then placed into 0.15% BSA in Ringer solution containing a known concentration (0.90 ± 0.11 ng/μL; 163 ± 26 Bq/μL) of radiolabeled antibody from the same batch that was injected into the mice. Dialysate was collected until the fluid recovery was stabilized and the three following samples with a stable fluid recovery (97–103%) were used to calculate the probe recovery. The in vitro probe recovery for each microdialysis probe was defined as the ratio of the concentration in the dialysate (C_dialysate_) over the concentration in the external medium (C_ext_) as previously described by [30]:

$$In\,vitro\,probe\, recovery = \frac{{C_{dialysate} }}{{C_{ext} }}$$

Dialysate concentrations were converted into ISF concentrations (C_ISF_) by dividing the dialysate concentration (C_dialysate_) by the in vitro probe recovery of each microdialysis probe:

$$C_{ISF} = \frac{{C_{dialysate} }}{probe\,recovery}.$$

### Ex vivo biodistribution

The brain was immediately frozen on dry ice after the perfusion. Terminal blood samples were centrifuged at 10 000 × g for 5 min to obtain plasma. Radioactivity in the brain, whole blood, plasma, and pellet was measured in a gamma counter (2480 Wizard™, Wallac Oy PerkinElmer, Turku, Finland), and radioactivity concentration was quantified as the percent of injected dose corrected for body weight of the animal (%ID/g/bw) as described in [40].

### Autoradiography

Coronal sections (20 μm) of the probe area in the brain were prepared using a cryostat (CM1850, Leica Biosystems, Nussloch, Germany or NX70, Thermo Fisher Scientific), and the sections were mounted on glass slides (SuperFrost Plus, Thermo Fisher). The placement of the probe in the hippocampus was confirmed during the sectioning. The sections were exposed to a phosphor imaging plate (Fujifilm, Tokyo, Japan) for 7 days, together with a standard of ^125^I with known radioactivity. The imaging plates were scanned with an Amersham™ Typhoon™ Biomolecular Imager (GE Healthcare, Chicago, IL, USA) at 600 dots per inch. The generated digital image was converted with a lookup table (Royal) in ImageJ. The radioactivity standards were used to normalize intensities for images obtained from different plates except for the free ^125^I-injected brain, where intensity was increased, since the radioactivity was too low to be detected on the same scale as antibody-injected brains.

### Immunohistochemistry

Immunohistochemistry was used to study microglia (ionized calcium binding adaptor molecule 1, Iba1) and astrocytes (glial fibrillary acidic protein, GFAP) on brain sections prepared from the mice that had undergone microdialysis. Sections were fixed in 4% paraformaldehyde (PFA) for 30 min, and then the antigen was retrieved by incubation in preheated citrate buffer (25 mM, pH 6.3) in microwave for 10 s. Sections were then allowed to reach RT for 30 min. Sections were permeabilized with 0.4% Triton-X 100 in PBS for 5 min, and then the primary antibodies (1:200 Iba1, ab178846, Abcam, Cambrigde, UK) and (1:400 GFAP, M0761, Agilent Dako, Santa Clara, CA, USA) in 0.1% Tween in PBS were incubated overnight in + 4 °C. Secondary antibodies goat anti-rabbit (1:500 Alexa fluor 488, A11008, Sigma-Aldrich) and goat anti-mouse (1:500 Alexa fluor 555, A21424, Sigma-Aldrich) in 0.1% Tween20 in PBS were incubated for 30 min, prior to mounting the slides with Vectashield^®^ Antifade Mounting Medium with DAPI (Vector Laboratories, Burlingame, CA, USA). The slides were washed with PBS between all incubations. The immunofluorescence staining was imaged with a Zeiss Observer Z.1 microscope and ZEN 2.6 software (Carl Zeiss Microimaging GmbH, Jena, Germany). In addition to glial markers, Aβ pathology was investigated with Aβ42 immunohistochemistry and Thioflavin-S staining according to previously published protocols [23].

### Thin layer chromatography

Thin layer chromatography (TLC) was used to confirm that ^125^I remained attached to the antibody. Dialysates 4–6 h post-injection were pooled together, and 3 × 5 ul was pipetted on a TLC filter paper. 300 Bq of ^125^I was pipetted on a control slide and ^125^I standards on a separate filter paper. Filter papers (excluding the standards) were placed into a chamber containing 70% acetone to separate free ^125^I and intact ^125^I-labeled antibody. The filter papers were then exposed to a phosphor imaging plate (MS, Multisensitive, PerkinElmer, Downers Grove, IL, USA) for 7 days. The imaging plates were scanned in a Cyclone Plus phosphor imager (PerkinElmer) at 600 dots per inch.

### Meso scale discovery

Meso Scale Discovery (MSD) was used to measure the concentration of mAb3D6 and mAb3D6-ScFv8D3 in the dialysate. MSD was performed on Standard Quickplex 96-well plates (Meso Scale Diagnostics, Rockville, MD, USA) coated with 1 μM Aβ1-42 protofibrils (Innovagen AB, Lund, Sweden) in PBS overnight at + 4 °C. Coated plates were blocked with 1% MSD Blocker A solution (Meso Scale Diagnostics) for 1 h at RT. All further dilutions were made in 1% MSD Blocker A solution. For standards, 0.1 μg/mL mAb3D6 and mAb3D6-scFv8D3 were fivefold serially diluted. Dialysates 4–6 h post-injection were pooled together and diluted 1:1. Samples were pipeted on the 96-well plate as duplicates (2 × 50 μL) and incubated in a shaker for 2 h at RT. Secondary antibody was biotinylated horse anti-mouse IgG (dilution 1:1000, BA-2000, Vector Laboratories), which was then detected with sulfo-tag labeled streptavidin (dilution 1:1000, Meso Scale Diagnostics), both incubated in a shaker for 1 h at RT. MSD Read Buffer T was added and the plate was read with MSD SECTOR Imager (Meso Scale Diagnostics). The results were analyzed using MSD Discovery Workbench software.

### Statistics

Data are presented as mean ± standard error of the mean. One-way analysis of variance (ANOVA) followed by Tukey's post hoc test was performed in GraphPad Prism 9.3.1 (GraphPad Software Inc., San Diego, CA, USA) and repeated measures ANOVA in IBM SPSS Statistics 28.0.1.0. (IBM Corporation, Armonk, NY, USA). One mouse was removed from the microdialysis data analysis as an outlier, since ISF %ID/g/bw values were above the criteria mean ± SD × 2 indicating a non-functional probe.

## Results

### In vitro affinity after radiolabeling

Radiolabeling did not cause any significant effect on the binding to the antigens (Aβ and TfR) measured by ELISA. The binding affinity to Aβ was similar for radiolabeled and non-radiolabeled antibodies. The binding affinity to murine TfR (mTfR) was slightly decreased after radiolabelling, but the difference was not statistically significant (Additional file 1: Fig. S1). The results were similar to previous data [40]. The specific activity was 218 ± 46 and 186 ± 35 Bq/ng for [^125^I]I-mAb3D6 and bispecific [^125^I]I-mAb3D6-scFv8D3, respectively.

### Pharmacokinetics in the ISF measured by microdialysis

**Fig. 2:**
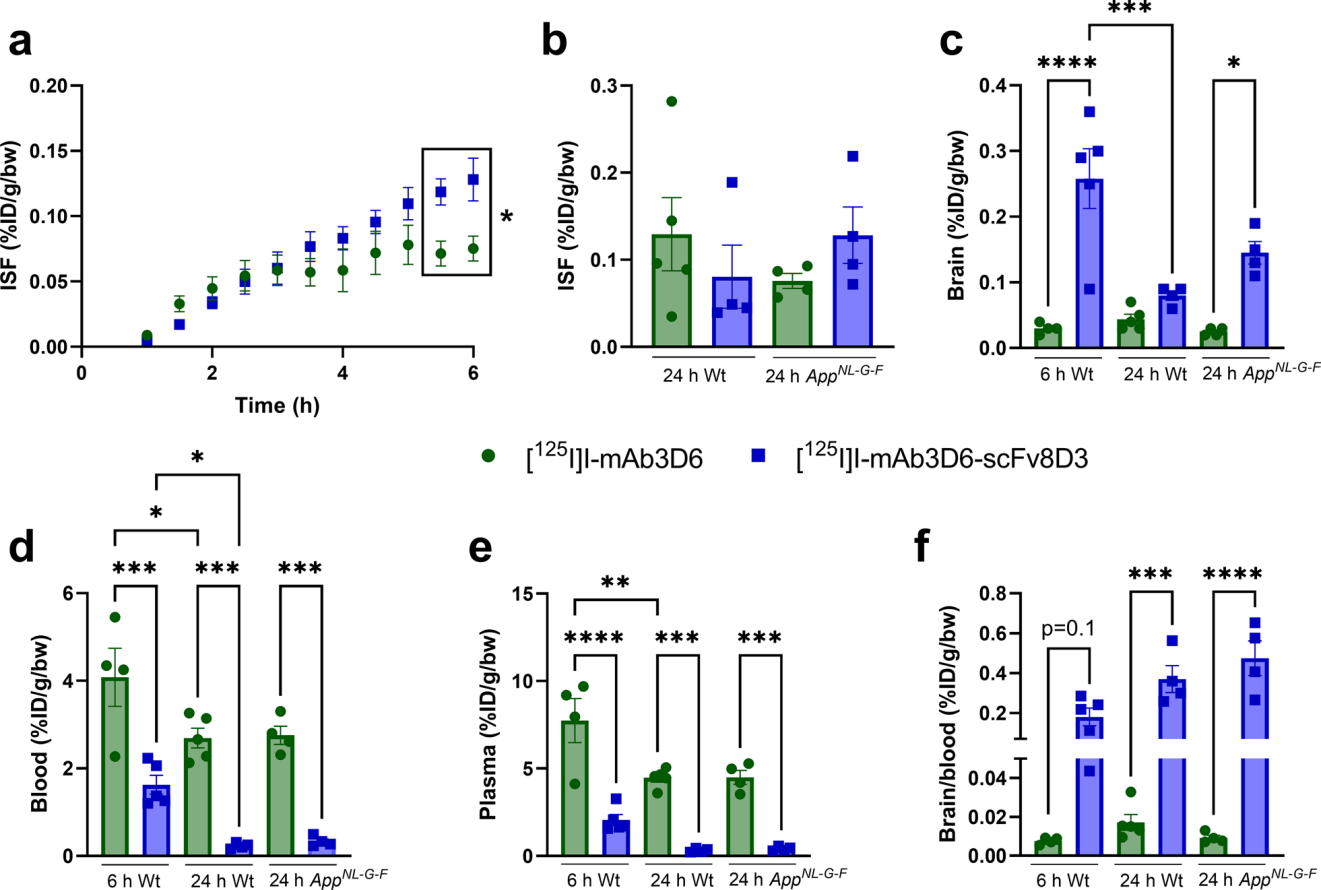
ISF dialysate concentration of [^125^I]I-mAb3D6 and [^125^I]I-mAb3D6-scFv8D3 in the hippocampus was measured by microdialysis 0–6 h (**a**) or 24 h (**b**) post-injection. The concentration of [^125^I]I-mAb3D6-scFv8D3 and [^125^I]I-mAb3D6 was measured 6 h post-injection in wild-type (Wt) mice and 24 h post-injection in Wt and in App^NL−G−F^ mice in the brain (**c**), blood (**d**) and plasma (**e**). Brain to blood ratio is presented in **f**. Data is presented as mean ± SEM. **a** Repeated measures ANOVA; **b**–**f** one-way ANOVA with Tukey's post-hoc test. *p < 0.05, **p < 0.01, ***p < 0.001, ****p < 0.0001. n = 4–5 mice/group

Microdialysis experiments demonstrated that both monospecific [^125^I]I-mAb3D6 and bispecific [^125^I]I-mAb3D6-scFv8D3 entered the brain and that the antibodies could be measured in the ISF in the hippocampus 1 h after the intravenous injection. The ISF concentration of [^125^I]I-mAb3D6-scFv8D3 continued to increase during at least 6 h post-injection, while the concentration of [^125^I]mAb3D6 seemed to reach a plateau already between 2 and 3 h post-injection (Fig. 2a). The concentration of the antibodies was not increased at 24 h post-injection compared to the earlier measured timepoints (Fig. 2a, b). The bispecific [^125^I]I-mAb3D6-scFv8D3 displayed elevated ISF concentrations in the hippocampus compared to the monospecific [^125^I]I-mAb3D6 at 5.5–6 h after the intravenous injection, also indicating an increased difference between the two antibodies with time (Fig. 2a, *p* = 0.034). However, the concentrations were no longer significantly different at 24 h post-injection in either Wt or *App*^*NL−G−F*^ mice (Fig. 2b, Wt, *p* = 0.733; *App*^*NL−G−F*^*, p* = 0*.*721). TLC showed that radioactivity detected in the dialysate originated from intact ^125^I-labeled antibody as almost no free ^125^I was detected in the dialysate (Additional file 1: Fig. S2). This was supported by a control experiment in mice injected with free ^125^I instead of a radiolabeled antibody, which showed that free ^125^I in the ISF dialysate decreased rapidly after intravenous injection (Additional file 1: Fig. S3). Antibody concentrations in the dialysate were confirmed with MSD measurement that showed a good correlation with γ-counter measurement at high concentrations, but most of the in vivo dialysates had too low concentration to be reliably measured with MSD (Additional file 1: Fig. S4).

The in vitro probe recovery was varied slightly between the individual probes and was 4.3 ± 0.5% and 3.9 ± 0.6% for [^125^I]I-mAb3D6 and [^125^I]I-mAb3D6-scFv8D3, respectively. Two probes broke, when they were removed from the brain, and thus it was not possible to perform in vitro microdialysis for those probes. The in vivo concentrations for the two animals corresponding to these two probes were calculated based on the average in vitro probe recovery.

### Distribution of antibody in the brain tissue and blood

The total concentration of ^125^I-labeled antibodies in the brain and blood was studied by measurement of radioactivity in the tissues with a γ-counter, while the spatial brain distribution was studied by ex vivo autoradiography. The concentration of [^125^I]I-mAb3D6-scFv8D3 was higher than that of [^125^I]I-mAb3D6 in the brain tissue of Wt mice 6 h post-injection (*p* < 0.0001, Fig. 2c), but the difference was no longer significant at 24 h post-injection (*p* = 0.873), and the concentration of [^125^I]I-mAb3D6-scFv8D3 decreased from 6 to 24 h (*p* = 0.0003). Additionally, [^125^I]I-mAb3D6-scFv8D3 displayed a higher brain concentration than [^125^I]I-mAb3D6 in the *App*^*NL−G−F*^ mice 24 h post-injection (*p* = 0.024, Fig. 2c).

Blood concentration of [^125^I]I-mAb3D6-scFv8D3 was lower than the concentration of [^125^I]I-mAb3D6 at terminal time-points in Wt mice and *App*^*NL−G−F*^ mice (Wt at 6 h, *p* = 0.0001; Wt at 24 h, *p* = 0.0001; *App*^*NL−G−F*^ at 24 h, *p* = 0.0003, Fig. 2d). Blood concentration of both [^125^I]I-mAb3D6 and [^125^I]I-mAb3D6-scFv8D3 decreased from 6 to 24 h ([^125^I]mAb3D6, *p* = 0.035; [^125^I]mAb3D6-scFv8D3, *p* = 0.034, Fig. 2d). Plasma concentrations displayed a similar difference between [^125^I]I-mAb3D6 and [^125^I]I-mAb3D6-scFv8D3 as the blood concentrations (Wt at 6 h, *p* = 0.0001; Wt at 24 h, *p* = 0.0002; *App*^*NL−G−F*^, *p* = 0.0005, Fig. 2e). The brain-to-blood concentration ratio was also elevated for [^125^I]I-mAb3D6-scFv8D3 compared to [^125^I]I-mAb3D6 in Wt (*p* = 0.0003) and *App*^*NL−G−F*^ (*p* < 0.0001) mice 24 h post-injection, and a similar trend was found 6 h post-injection (*p* = 0.123) (Fig. 2f). The ISF-to-blood (Wt, *p* = 0.0201; *App*^*NL−G−F*^, *p* = 0.0023) and the ISF-to-plasma ratios (Wt, *p* = 0.0062; *App*^*NL−G−F*^, *p* = 0.0003) were also higher for [^125^I]I-mAb3D6-scFv8D3 compared to [^125^I]I-mAb3D6 at 24 h post-injection (Additional file 1: Fig. S5).

**Fig. 3:**
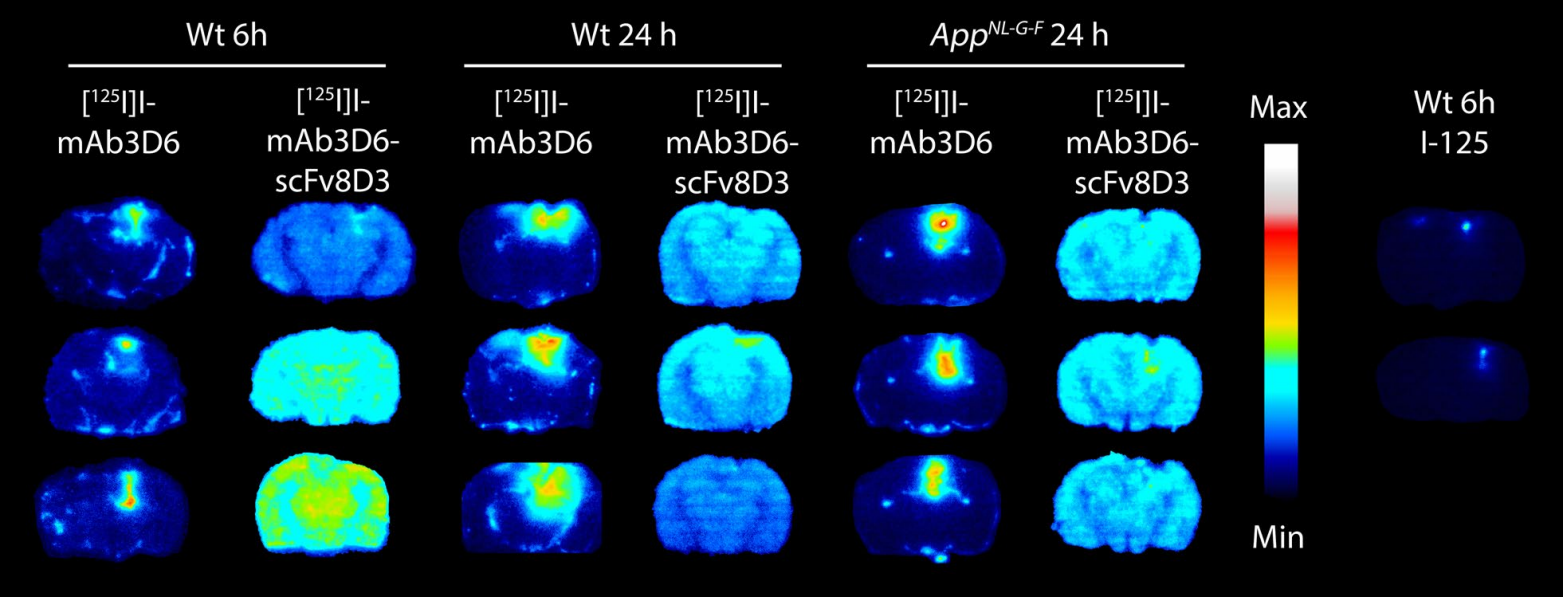
Coronal brain sections of three representative individuals per group euthanized at the end of the microdialysis experiment, i.e. 6 h or 24 h after the intravenous injection of [^125^I]I-mAb3D6, [^125^I]I-mAb3D6-scFv8D3 or ^125^I

Ex vivo autoradiography showed that [^125^I]I-mAb3D6 was mainly present in the probe area in the hippocampus and the surrounding areas of the brain in Wt mice 6 h and 24 h post-injection and in *App*^*NL−G−F*^ mice 24 h post-injection, while this distribution pattern was not observed for [^125^I]I-mAb3D6-scFv8D3 that was evenly observed in the whole brain in all groups (Fig. 3). Additionally, in brain sections prepared from mice injected with free ^125^I, radioactivity was detected around the probe area, although the total signal was very much lower compared to the signal detected in the probe area of the mAb3D6-injected mice (Fig. 3). Immunohistochemistry and ThS staining confirmed abundant Aβ pathology in the *App*^*NL−G−F*^ mice, while no Aβ aggregates were found in the Wt mice (Additional file 1: Fig. S6).

### Glial cell markers Iba1 and GFAP around the probe area

**Fig. 4:**
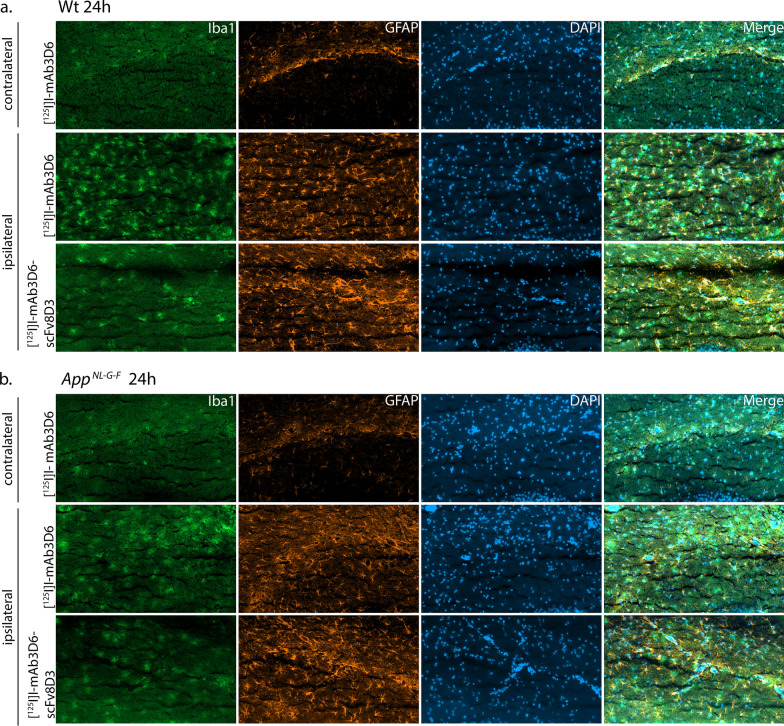
Iba1 and GFAP staining of the intact hippocampus contralateral to the probe and in the close proximity to the microdialysis probe in the hippocampus in wild-type (Wt) (**a**) and App^NL−G−F^ mice (**b**) that were perfused 24 h after an intravenous injection of [^125^I]I-mAb3D6 or [^125^I]I-mAb3D6-scFv8D3

Proteins Iba1 (microglia) and GFAP (astrocytes) were studied around the probe area in the brain by immunohistochemistry to detect potential immune responses caused by the guide cannula surgery and probe insertion, and to reveal effects related to immunotherapy, which could explain the difference in the brain distribution of the two antibodies. Iba1 and GFAP staining was detected in close proximity to the probe area in the hippocampus and the surrounding areas of the brain. In contrast, there were only few astrocytes and microglia stained in the intact hippocampi contralateral to the probe (Fig. 4 and Additional file 1: Fig. S7). The position of the images in relation to the probe is shown in the supplementary (Additional file 1: Fig. S7). Since the immune response was the same for both antibodies, it indicated that the insertion of the microdialysis probe caused the response and that this was unrelated to the format of the antibody used.

## Discussion

The present study investigated the brain distribution of monospecific mAb3D6 and bispecific mAb3D6-scFv8D3 in the brain ISF by microdialysis, and in the brain tissue and in the blood by ex vivo gamma-counting and ex vivo autoradiography. Microdialysis showed that the ISF concentration of [^125^I]I-mAb3D6-scFv8D3 was higher than that of [^125^I]I-mAb3D6, but the difference was only approximately twofold at 6 h post-injection in Wt mice although the total concentration difference in the brain tissue was approximately eightfold, indicating that a larger proportion of bispecific [^125^I]I-mAb3D6-scFv8D3 was associated with brain tissue compared to the monospecific [^125^I]I-mAb3D6. A previous study has demonstrated that a significant fraction of [^125^I]I-mAb3D6-scFv8D3 that is associated with brain tissue at this time point after injection is bound to the capillaries in the brain [40], most likely to TfR expressed by the endothelial cells. In addition, it has been shown that TfR is expressed by neurons [15, 41], which could be a potential binding site for TfR antibodies once they have crossed the BBB. Thus, binding of [^125^I]I-mAb3D6-scFv8D3 to TfR on endothelial cells and neurons could explain why the difference in total brain concentration between the bispecific and monospecific antibody was higher than the difference observed in the ISF concentration. Interestingly, the ISF concentrations of the two antibodies were similar in Wt and *App*^*NL−G−F*^ mice at 24 h post-injection, suggesting that antibody interactions with Aβ deposits, which were confirmed for the *App*^*NL−G−F*^ mice included in the study, had a minor effect on the concentration of free antibody. It has also previously been shown by Western blot analysis of whole brain homogenates that TfR levels are similar in Wt mice and Aβ expressing mice, although it should be noted that another AD mouse model (3xTg model) was used in this previous study and that the mice were somewhat older than those used in the present study [42]. Since whole brain samples were used in the analysis, the measured TfR levels represent both TfR expressed by endothelial cells and other cell types such as neurons. Thus, it cannot be completely excluded that TfR levels at the BBB and in the brain parenchyma may be influenced by the presence of Aβ pathology, and subsequently that it could impact the brain pharmacokinetics of the bispecific antibody.

In addition, the brain-to-blood, ISF-to-blood and ISF-to-plasma ratios of [^125^I]I-mAb3D6-scFv8D3 were significantly higher compared to mAb3D6, indicating more efficient delivery across the BBB for the bispecific antibody. Ex vivo autoradiography revealed that the fusion of a TfR binding-moiety to mAb3D6 had a remarkable effect on the brain distribution of the antibody, as [^125^I]I-mAb3D6-scFv8D3 was distributed evenly in the whole brain, while [^125^I]I-mAb3D6 was mostly distributed around the probe area similarly to the radionuclide ^125^I in the control experiment albeit at a much higher concentration. The global brain distribution of antibodies has not been studied in the previous publications where antibodies have been measured in the brain ISF by microdialysis [30, 32]. As the present study shows the distribution is affected by the guide cannula and probe insertion, it may indicate that previous microdialysis studies may have over-estimated the average antibody ISF concentrations, i.e. the concentrations seen in an intact brain. This could also mean that the global ISF concentration of the monospecific antibody in the present study could be lower than that measured by microdialysis close to the probe, and thus, that the difference between the monospecific and the bispecific antibodies in fact is larger than the twofold difference detected in the present study. The reason for the difference in concentration between the mono- and bispecific antibody in this regard may be attributed their different mechanisms of brain entry. The bispecific antibody enters the brain after engaging with the TfR on endothelial cells throughout the whole brain capillary network. Thus, the antibody is rapidly distributed to the whole brain volume and at the same time, interactions with peripheral and blood cell expressed TfR keeps plasma concentration low, reducing the amount of antibody that can leak through around the probe insertion site. In contrast, free plasma concentrations of the monospecific antibody are high, which promotes leakage through damaged vessels. Further, it has been proposed that antibodies enter the brain through a perivascular route rather than through the BBB [43]. Thus, even if damage caused by the probe does not entirely rupture a vessel, its perivascular portion could be damaged and induce increased entry of the antibody. In addition to mechanical BBB disruption, it has been debated to what extent the insertion of the guide cannula causes neuroinflammation, and the timing of this event. In the present study the guide cannula was inserted a week before the microdialysis experiment to allow for the BBB to be at least partly restored and for the acute inflammation reaction to cease. However, an increased level of glial cell markers GFAP and Iba1 was still detected in the probe area at this time point indicating inflammatory processes that could potentially also lead to a more “leaky” BBB.

To our knowledge, there are only few other research groups who have measured antibody concentration in the brain ISF in rodents by using microdialysis [30, 32]. The microdialysis method is time-consuming and measuring large molecules such as antibodies by microdialysis is much more complicated than measuring small molecules. One of the main challenges is that large pores on the semipermeable membrane of the probe can easily cause ultrafiltration of perfusion fluid into the tissue. This challenge can be solved by using a push-and-pull microdialysis system, adding albumin to the perfusion fluid to increase osmotic pressure, and by continuously measuring fluid recovery and adjusting peristaltic pump flow rate to keep the fluid recovery stable as in the present and previous studies [30, 44]. A possible solution to prevent ultrafiltration even more efficiently could be automated fluid recovery monitoring that was introduced in the study by Le Prieult et al. [32].

Measuring in vivo probe recovery would be the most optimal way to estimate the actual ISF concentration of the antibody based on the concentration in the dialysate, but in our setup with a radiolabeled antibody, it was not possible to measure in vivo probe recovery without contaminating the microdialysis equipment with ^125^I that has a relatively long half-life. Studies comparing the correlation between in vitro and in vivo probe recovery for small molecules have been controversial and in vivo probe recovery is dependent on the conditions in the tissue [45, 46]. There is only limited data available of in vitro* and *in vivo probe recovery for large molecules, but the in vitro and in vivo recovery of a therapeutic monoclonal antibody in peripheral tissues were similar in the study by Jadhav et al. [47] and Takeda et al. [48].

Another challenge in measuring antibodies in the brain ISF is the low probe recovery and low concentration of antibody in the ISF, and thus also the low concentration of antibody in the dialysate. In the present study, the antibody dose was relatively low, making it even more challenging to measure the antibody in the dialysate. Radiolabeling of the antibodies allowed for the measurement of the antibody concentration in the dialysate relatively reliably, but the lowest concentrations in the dialysate were close to the detection limit of the γ-counter. The antibody concentration in the dialysate was also confirmed by MSD measurement. Although the sensitivity was too low for most of the in vivo dialysates, there was a good correlation between the γ-counter and MSD results for samples in the higher concentration range, which further supports the use of radioactivity to measure antibody concentrations. LC–MS/MS or another more sensitive method could be considered to verify the low antibody concentrations in the ISF in future studies. One possibility to improve probe recovery and to increase antibody concentration in the dialysate would be to use open flow microperfusion that allows free convection flow through macroscopic openings of the probe instead of semipermeable membrane used in microdialysis. This method has been successfully used to measure antibodies or nanobodies in the hippocampus in mice [32, 49]. However, open-flow microperfusion is very sensitive to changes in fluid recovery and requires continuous monitoring of flow rate. In vitro recovery measurement is also complicated.

Improving brain access by using bispecific antibodies could provide significant improvement to current immunotherapies. Better understanding of antibody pharmacokinetics in the brain is an essential step to develop safe, effective and reasonably priced antibody treatments for AD.

## Conclusions

In conclusion, the present study showed that microdialysis is a suitable method to study brain pharmacokinetics of monospecific and bispecific radiolabeled antibodies, although there are several challenges such as low probe recovery, low antibody concentration in the dialysate, and elevated antibody distribution and inflammation around the probe area in the brain. The bispecific antibody crossed the BBB better than monospecific antibody and appeared to be distributed or associated with brain tissue to a higher degree than its monospecific version.

## Supplementary Information

**Additional file 1. Additional file of Brain pharmacokinetics of mono- and bispecific amyloid-β antibodies in wild-type and Alzheimer's disease mice measured by high cut-off microdialysis.**

## Data Availability

The datasets used and/or analyzed during the current study are available from the corresponding author on reasonable request.

## Abbreviations

Aβ: Amyloid beta
AD: Alzheimer's disease
BBB: Blood–brain barrier
BSA: Bovine serum albumin
CNS: Central nervous system
CSF: Cerebrospinal fluid
FEP: Fluorinated ethylene propylene
ISF: Interstitial fluid
mAb: Monoclonal antibody
PET: Positron emission tomography
ScFv: Single-chain variable fragment
Wt: Wild-type
