# Supplementary material for: Brain pharmacokinetics of mono- and bispecific amyloid-β antibodies in wild-type and Alzheimer’s disease mice measured by high cut-off microdialysis
Source: Fluids Barriers CNS. 2022 Dec 12;19:99. doi: 10.1186/s12987-022-00398-w (PMC9743601; doi:10.1186/s12987-022-00398-w)
Supplement: Supplementary file 1 — Additional file 1. Additional file of Brain pharmacokinetics of mono- and bispecific amyloid-β antibodies in wild-type and Alzheimer's disease mice measured by high cut-off microdialysis. [file 12987_2022_398_MOESM1_ESM.docx]

## Supplementary material

Fig. S 1 Representative figures for Aβ protofibril and mTfR ELISAs showing that radiolabeling did not significantly affect the binding affinity of mAb3D6 and mAb3D6-scFv8D3.


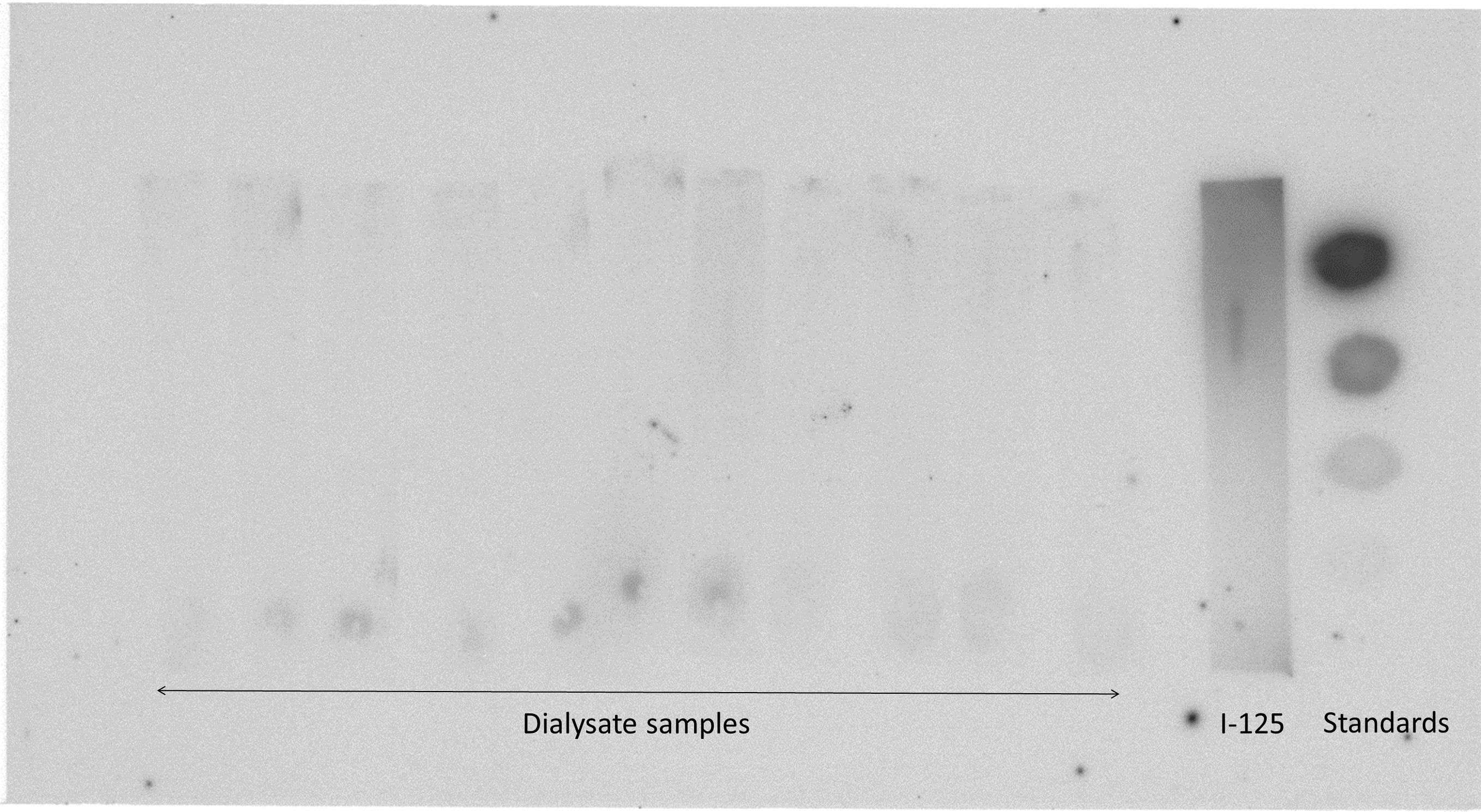


I-125

Standards

Fig. S 2 Thin layer chromatography of dialysates. Each dialysate sample is from a different animal.

Fig. S 3 ISF concentration of ^125^I was measured by microdialysis 0-6 h after an intravenous injection of ^125^I in Wt mice (n = 2).

Fig. S 4 Correlation of the antibody concentration in the dialysate when measured by MSD or γ-counter.

Fig. S 5. ISF-to-blood (a) and ISF-to-plasma (b) ratio 6 h and 24 h after intravenous injection of mAb3D6 or mAb3D6-scFv8D3.


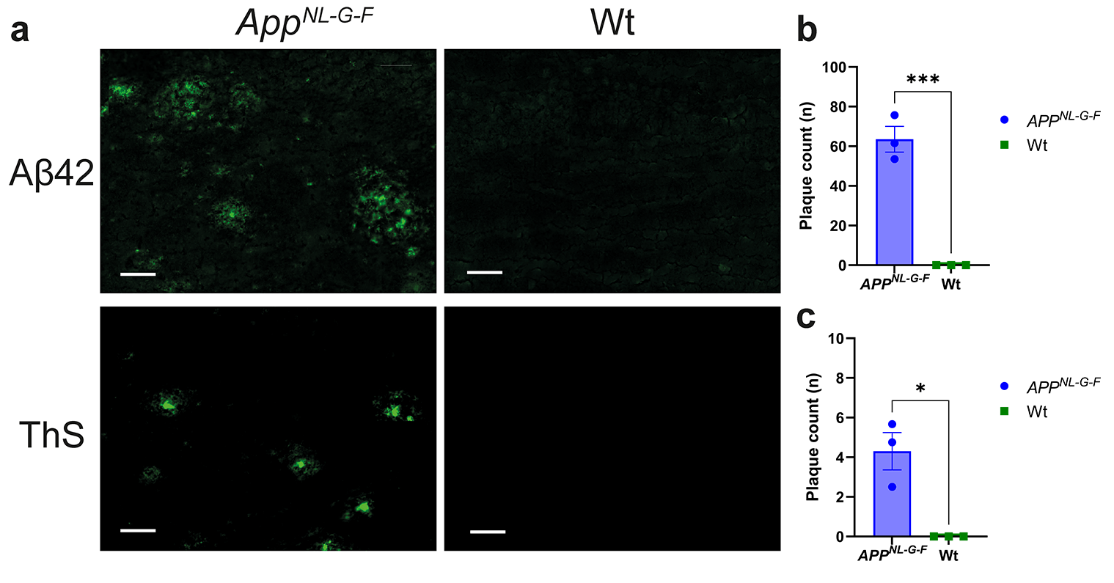


Fig. S 6. Aβ pathology in AppP^NL-G-F^ and Wt mice visualized by Aβ42 immunohistochemistry and Thioflavin-S (ThS) staining (a). Clear differences were observed between the genotypes and abundant pathology was present in the 8 months old App^NL-G-F^ mice used in the study. Also in line with previous reports, the pathology was dominated by Aβ42 and mainly diffuse, with small ThS positive cores. Number of Aβ42-positive (b) and ThS-positive (c) plaques based on 3-4 images per animal (n = 3 each group) and shown as mean ± SEM, *p < 0.05, ***p < 0.001. Scale bar: 50 μm.


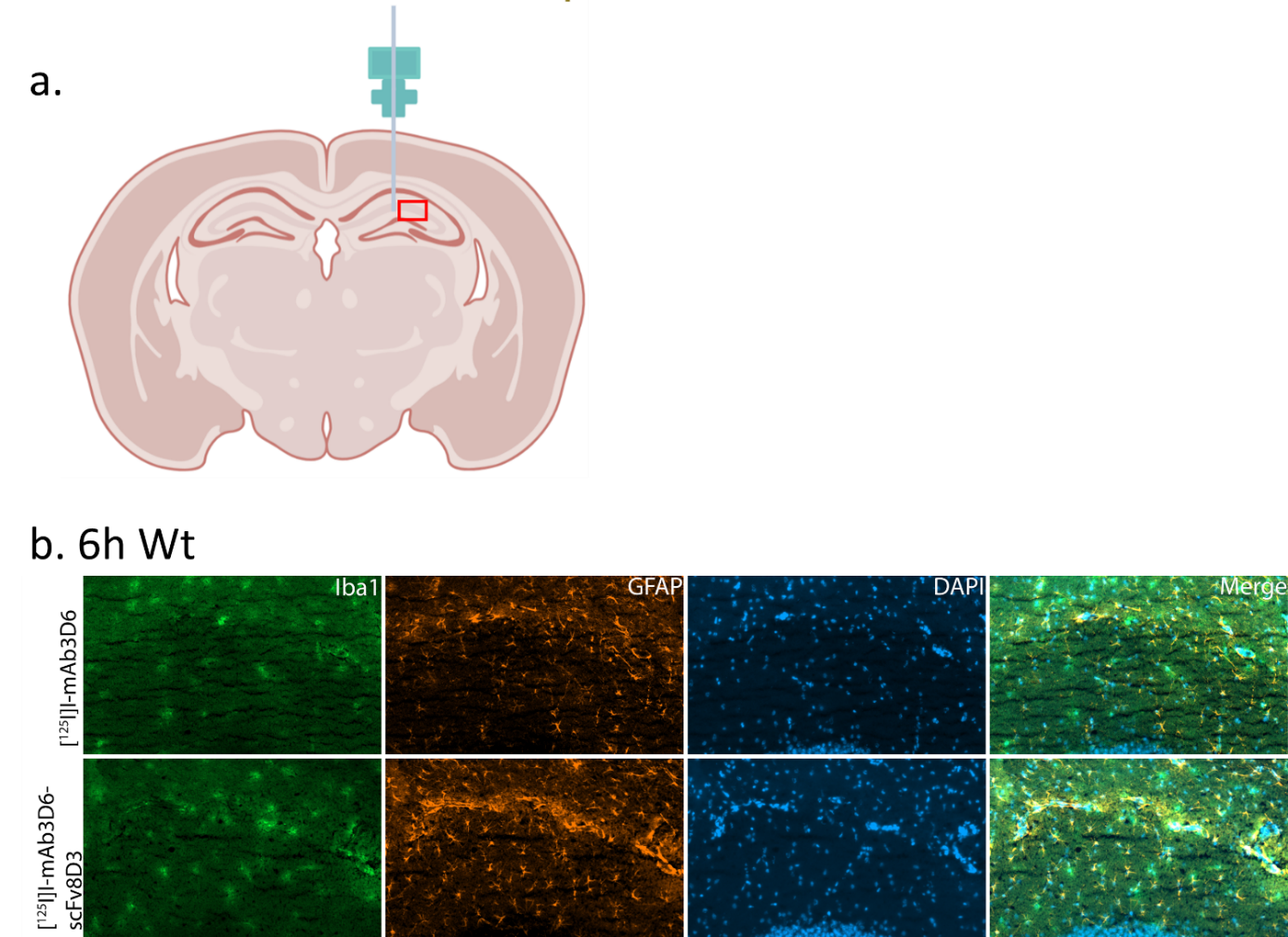


Fig. S 7. Position (red box) of the images showing Iba1 and GFAP staining in relation to the microdialysis probe (a). Iba1 and GFAP staining in the close proximity to the microdialysis probe in the hippocampus in wild-type (Wt) (b) that were perfused 6 h after an intravenous injection of [^125^I]I-mAb3D6 or [^125^I]I-mAb3D6-scFv8D3.
